# Transient early food restriction leads to hypothalamic changes in the long-lived crowded litter female mice

**DOI:** 10.14814/phy2.12379

**Published:** 2015-04-23

**Authors:** Marianna Sadagurski, Taylor Landeryou, Gillian Cady, Andrzej Bartke, Ernesto Bernal-Mizrachi, Richard A Miller

**Affiliations:** 1 Division of Geriatric and Palliative Medicine, Department of Internal Medicine, University of MichiganAnn Arbor, Michigan; 2 Department of Pathology and Geriatrics Center, University of MichiganAnn Arbor, Michigan; 3 Department of Internal Medicine–Geriatrics Research, Southern Illinois University School of MedicineSpringfield, Illinois; 4 Division of Metabolism Endocrinology and Diabetes, Department of Internal Medicine, University of MichiganAnn Arbor, Michigan; 5 Endocrinology Section, Medical Service, Veterans Affairs Medical CenterAnn Arbor, Michigan

**Keywords:** Aging, caloric restriction, hypothalamus, metabolism

## Abstract

Transient nutrient restriction in the 3 weeks between birth and weaning (producing “crowded litter” or CL mice) leads to a significant increase in lifespan and is associated with permanent changes in energy homeostasis, leptin, and insulin sensitivity. Here, we show this brief period of early food restriction leads to permanent modulation of the arcuate nucleus of the hypothalamus (ARH), markedly increasing formation of both orexigenic agouti-related peptide (AgRP) and anorexigenic proopiomelanocortin (POMC) projections to the paraventricular nucleus of the hypothalamus (PVH). An additional 4 weeks of caloric restriction, after weaning, does not further intensify the formation of AgRP and POMC projections. Acute leptin stimulation of 12-month-old mice leads to a stronger increase in the levels of hypothalamic pStat3 and cFos activity in CL mice than in controls, suggesting that preweaning food restriction leads to long-lasting enhancement of leptin signaling. In contrast, FoxO1 nuclear exclusion in response to insulin is equivalent in young adult CL and control mice, suggesting that hypothalamic insulin signaling is not modulated by the crowded litter intervention. Markers of hypothalamic reactive gliosis associated with aging, such as Iba1-positive microglia and GFAP-positive astrocytes, are significantly reduced in CL mice as compared to controls at 12 and 22 months of age. Lastly, age-associated overproduction of TNF-*α* in microglial cells is reduced in CL mice than in age-matched controls. Together, these results suggest that transient early life nutrient deprivation leads to long-term hypothalamic changes which may contribute to the longevity of CL mice.

## Introduction

Calorie restriction (CR) extends longevity in most rodent species (Bartke et al. 2002). Recent work from our laboratory showed that transient reduction in food availability limited to the period between birth and weaning (producing “crowded litter” (CL) mice) increased the lifespan of genetically normal mice (Sun et al. 2009) and led to long-lasting changes in metabolic status including reduced body weight, improved energy balance, glucose homeostasis, and leptin sensitivity (Sadagurski et al. 2014). However, the site of action responsible for the effect in the CL system is not clear. The central nervous system (CNS) is sensitive to changes in early postnatal environment, particularly during a critical window of hypothalamic development. An abnormal hormonal milieu during development can trigger persistent changes in the function of hypothalamic neurocircuits, which lead to long-lasting effects on the body's energy balance (Plagemann et al. 2012).

The important components of this network include neurons located in the arcuate nucleus of the hypothalamus (ARC), and particularly neurons which produce proopiomelanocortin (POMC), the anorectic regulators, and neuropeptide Y (NPY)/agouti-related peptide (AgRP), the major orexigenic signals (Bouret and Simerly 2006). Each of these neuronal populations provides overlapping projections to other key parts of the hypothalamus, including the paraventricular nucleus of the hypothalamus (PVH) (Bouret 2009). ARC neural circuits involved in appetite regulation develop primarily during the first 3 weeks of postnatal life in rodents, under the control of both genetic and environmental factors (Bouret 2010). In this context, rearing rats in large litters enhances early life leptin sensitivity and the development of the ARC-PVN pathway, enhancing both the catabolic *α*-MSH and anabolic AgRP projections to the PVH (Patterson et al. 2010). In contrast, AgRP innervations onto POMC neurons significantly increase with age or as a consequence of chronic high-fat diet (HFD) feeding, thus reducing neuronal activity in POMC neurons (Bouret et al. 2008; Newton et al. 2013). Similarly, maternal HFD feeding during lactation predisposes the offspring for impairment of the hypothalamic melanocortin circuitry (Vogt et al. 2014).

Postnatal CR associated with reduced body weight alters hypothalamic leptin receptor (LepRb) signaling and neuropeptide balance, thus changing the net energy balance of older animals (Coupe et al. 2012). Similarly, advanced age significantly reduces LepRb protein expression and the downstream signaling pathway (Bigford et al. 2012). In addition to leptin, insulin also appears to exert important influences on the development of hypothalamic circuits that regulate energy homeostasis (Bouret 2009).

Chronic food restriction in rodents delays the onset and incidence of age-related diseases, and retards many other sequelae of aging (Martin et al. 2006). Aging retardation can also be achieved in mice by inhibiting activation of IkB kinase-*β* (IKK-*β*) and nuclear factor kB (NF-kB) inflammatory pathways in the hypothalamus (Zhang et al. 2013). Conversely, a constitutively active expression of inflammatory IKK-*β* in the hypothalamus reduces both insulin and leptin signaling, whereas administration of an IKK*β* inhibitor reverses HFD-induced hypothalamic insulin resistance (Purkayastha et al. 2011). Thus, alterations in hypothalamic function may be of fundamental importance in the regulation of aging and age-related diseases (Tang and Cai 2013). In this study, we evaluated the effect of CL on hypothalamic development and age-associated hypothalamic inflammatory responses, to uncover possible developmental changes that might lead to the lifespan extension seen in CL mice.

## Methods

### Animals

Procedures involved in this study were approved by the University Committee on the Use and Care of Animals (UCUCA) of the University of Michigan. Mice from the genetically heterogeneous UM-HET3 stock were produced as previously described (Miller et al. 1999). These mice provide several advantages compared to inbred mice often used for physiological analyses, because their genetic heterogeneity reduces the likelihood that findings might reflect the idiosyncrasies of a specific, and entirely homozygous, genotype. This genetic heterogeneity is a better approximation of heterogeneity that would be seen in a population of noninbred humans, and the use of UM-HET3 mice for many other studies of longevity and physiology in mice has provided an extensive collection of helpful background data as well (Miller et al. 2007, 2011; Steinbaugh et al. 2012; Sadagurski et al. 2014). Litters were culled to eight, after which either 0, 4, or 7 additional pups were added, from another litter, thus leading to total litter size of 8 (CL8, used as controls), 12 (CL12), or 15 (CL15) pups. Litters were produced by six foster mothers. At 23 days of age, female pups were weaned into cages containing four mice per cage, and maintained thereafter with free access to normal chow. Only female mice were used for the work described in this study: we used approximately 20 cages. Mice were killed at 6 weeks, 24 weeks, 12 months, and 22 months of age. Estrous cycle was not taken into consideration in this study. The males were left in the litters, although not used and that the sex ratio was not adjusted. All mice were ad lib on Purina 5001, (23% protein and 6% fat) from 23 days until 4 weeks old. For CR feeding: from 4 weeks of age, CR mice were given 80% of the amount of food consumed by age-matched ad lib control mice. From 6 weeks old and continuing until euthanasia at 8–9 weeks, the CR mice were given 60% of diet consumed by ad lib control mice. Mice on CR feeding were killed at 8–9 weeks old. The amount of food the CR mice was given was determined by measuring food consumption, over a 5-day period each week, for three female CL8 cages and three female CL12 cages, set up 1–2 weeks prior to the first cage of mice to be subjected to CR. For CR experiments we used eight cages of females. The average amount of food eaten per mouse was calculated at each age and the appropriate percentage taken to determine what each cage of CR mice should be given daily. CR mice were fed daily based on the number of mice in the cage. Food was placed in between 7 am and 9 am every day.

### RNA extraction and qPCR

Hypothalami were carefully dissected using Brain Matrices (Braintree Scientific, Braintree, MA). Isolated mRNA from this tissue was analyzed using quantitative real-time PCR. RNA was isolated using the QIAGEN RNeasy Kit (QIAGEN, Valencia, CA), which was combined with the RNase-Free DNase Set (QIAGEN). RNA was reversely transcribed with High Capacity cDNA RT Kit and amplified using TaqMan® Universal PCR-Master Mix, NO AmpErase UNG with TaqMan® Assay-on-demand kits (Applied Biosystems, Foster City, CA). Relative expression of target mRNAs was adjusted for total RNA content by beta-actin RNA quantitative PCR. There were no statistically significant differences in beta-actin mRNA levels among any of the groups in the study. Calculations were performed by a comparative method (2−ΔΔCT). Quantitative PCR was performed on an ABI-PRISM 7900 HT Sequence Detection system (Applied Biosystems). Each reaction was carried out in triplicates as previously described (Sadagurski et al. 2010). *Il6* F: GTGGCTAAGGACCAAGACCA, *Il6* R: GGTTTGCCGAGTAGACCTCA, *Nfkbia* F: TGCCTGGCCAGTGTAGCAGTCTT, *Nfkbia* R: CAAAGTCACCAAGTGCTCCACGAT, *Ikbkb* F: GGCACCTTGGATGACCTAGA, *Ikbkb* R: CCATATCCTGGCTGTCACCT, *Ikbke* F: ACCACTAACTACCTGTGGCAT, *Ikbke* R: ACTGCGAATAGCTTCACGATG, *Tnfa* F: CATCTTCTCAAAACTCGAGTGACAA, *Tnfa* R: TGGGAGTAGATAAGGTACAGCCC, *Emr1* F: AATCGCTGCTGGTTGAATACAG, *Emr1* R: CCAGGCAAGGAGGACAGAGTT, *Cd68* F: CTTCCCACAAGCAGCACAG, *Cd68* R: AATGATGAGAGGCAGCAAGAGA, *AgRP* F: AGGGCA TCAGAAGGCCTGACCA, *AgRP* R: CTTGAAGAAGCGGCAGTAGCAC, *POMC* F: AAGAGCAGTGACTAAGAGAGGCCA, *POMC* R: ACATCTATGGAGGTCTGAAGCAGG.

### Intracerebral cannulation and insulin administration

As described previously (Leinninger et al. 2009) mice were anesthetized using an isoflurane vaporizer and placed in a stereotaxic frame (Kopf Instruments, Tujunga, CA). After exposing the skull and determining coordinates for bregma, a 26 g steel guide cannula with stylet (Plastics One, Roanoke, VA) was lowered toward the right lateral ventricle using the following coordinates from bregma: 0.6 mm posterior, 1.0 mm lateral, 2.1 mm ventral. The guide cannula was cemented to the skull using dental acrylic and the skin surrounding the cannula closed with sutures. After surgery, mice were singly housed and received Buprenex analgesia. Animals’ body weights and food intake were monitored daily, with additional daily handling that included removal and replacement of the stylet. Two weeks after surgery, and thus 24 h before experimental treatment, correct cannula placement was confirmed based on drinking response following angiotensin II injection (AGII; 10 *μ*A; American peptide, Sunnyvale, CA) diluted saline (injection volume: 1 *μ*) (Schwartz et al. 1992). On the day of treatment, animals were fasted for 4 h, followed by administration of insulin (300 mU, 2 *μ*L) or an equivalent volume of saline as described previously (Villanueva et al. 2009; Sadagurski et al. 2012). One hour after injection, animals were anesthetized by i.p. injection of Avertin (250 mg/kg) and perfused as detailed below.

For peripheral leptin treatment, mice were injected i.p. with either 5 mg/kg BW recombinant mouse leptin (1 mg) (provided by Dr. A Parlow, National Hormone and Pituitary Program, Torrance, CA) or vehicle as previously described (Sadagurski et al. 2012). 12-month-old female mice were killed 1 h after an i.p. injection of leptin or vehicle performed in overnight fasted animals.

### Perfusion and immunolabeling

Mice were anesthetized with an overdose of intraperitoneal (IP) pentobarbital and transcardially perfused with phosphate-buffered saline (PBS) (pH 7.5) followed by 4% paraformaldehyde (PFA). Brains were postfixed, dehydrated, then sectioned coronally (30 *μ*m) using a sliding microtome followed by immunohistochemical or immunofluorescent analysis as previously described (Patterson et al. 2010). For immunohistochemical labeling, free-floating brain sections were pretreated by sequential incubations in 0.3% H_2_O_2_/1% NaOH, 0.3% glycine, 0.03% SDS, followed by blocking in normal donkey serum (NDS). Sections were incubated in goat anti-AgRP (1:1000; Phoenix Pharmaceuticals, Belmont, CA), sheep anti-*α*-MSH (1:1000; Millipore, Temecula, CA), rabbit anti-FoxO1 (1:100; Cell Signaling, Danvers, MA), mouse anti-NeuN (1:1000; Cell Signaling), rabbit anti-pStat3 (1:1000; Cell Signaling), rabbit anti-cFos (1:500; Santa Cruz, Santa Cruz, CA), rabbit anti-GFAP (1:1000; Millipore), mouse anti-TNF*α* (1:500; Abcam, Cambridge, MA), and rabbit anti-Iba1 (1:1000; Wako, Richmond, VA) were used to detect primary antibodies followed by AlexaFluor-conjugated secondary antibodies (Invitrogen, Carlsbad, CA) as previously published (Munzberg et al. 2004; Bouret et al. 2008; Sadagurski et al. 2012; Zhang et al. 2013; Vogt et al. 2014). Sections were mounted onto Superfrost Plus slides (Fisher Scientific, Hudson, NH) and coverslipped with ProLong Antifade mounting medium (Invitrogen). For immunohistochemical labeling incubation in biotinylated donkey anti-rabbit (Jackson Immunoresearch) preceded avidin–biotin complex (Vectastain) and development with metal-enhanced DAB (ThermoScientific). Microscopic images were obtained using an Olympus FluoView 500 Laser Scanning Confocal Microscope (Olympus, Center Valley, PA) equipped with a 20× objective.

### Quantification analysis

The density of AgRP and *α*-MSH innervation of the PVN was determined by quantitative confocal microscopy using previously published methods (Patterson et al. 2010). For each animal, two sections through the anterior and posterior PVN were acquired (bregma-0.82 and −1.06). Image analysis was performed using Image J analysis software (version 1.39t; National Institutes of Health, Bethesda, MD). Each image plane was binarized to isolate the labeled fibers from the background as well as compensate for differences in fluorescence intensity and was then skeletonized so that each fiber segment was 1 pixel thick. The integrated intensity was then calculated for each image, which reflects the total number of pixels in the skeletonized image and was proportional to the total length of labeled fibers in the image. This procedure was carried out on each image plane in the stack, and the values for all image planes in a stack was summed. The resulting value is an accurate index of fiber density in the volume sampled (Patterson et al. 2010).

For quantification of immunoreactive-positive neurons, pictures of matched brain areas were taken from at least three sections containing the ARC of the hypothalamus for each brain between bregma −1.58 mm to −1.94 mm (according to the Franklin mouse brain atlas). To quantify astrocytosis, GFAP integrated intensity was calculated by correcting the values for the background using Image J (http://rsbweb.nih.gov/ij/). Serial brain sections across the MBH were made at 20 *μ*m thickness, and every five sections were represented by one section with staining and cell counting. All sections were arranged from rostral to caudal to examine the distribution of labeled neurons. The images were quantified with Imaris (versions 6.4 and 7.0; Bitplane), using the function spot to count nuclei and surface to measure the area or the volume of the different objects. The number of positive neurons was presented as means ± SEM.

### Statistical analysis

Data sets with more than two groups were analyzed using one-way analysis of variance (ANOVA) followed by Tukey's post hoc test. For statistical analyses of experiments involving insulin injections and inflammatory response, we performed two-way ANOVA followed by Tukey's post hoc test. Two-tailed Student's *t*-tests were used for comparisons involving only two groups. All data were presented as mean ± SEM. *P* < 0.05 was considered significant.

## Results

### Effects of CL on hypothalamic neurocircuits

**Figure 1:**
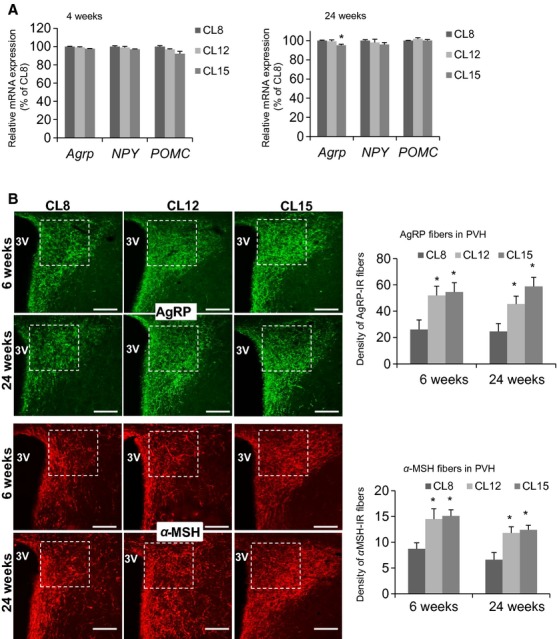
Effects of crowded litter (CL) on hypothalamic neurocircuits. (A) Quantitative real-time PCR analysis of hypothalamic agouti-related peptide (*Agrp*), neuropeptide Y (*NPY*), and proopiomelanocortin (*POMC*) mRNA expression upon 18 h fasting at 4 and 24 weeks of age of CL8 (control), CL12, and CL15 female mice; (*n* = 6/each group). (B) Images and quantification of agouti-related peptide (AgRP) and *α*-melanocyte-stimulating hormone (*α*-MSH) immunoreactive fibers innervating the paraventricular nucleus of the hypothalamus (PVH) at 6 and 24 weeks of age of CL8 (control), CL12, and CL15 female mice; (*n* = 6/each group). White boxes indicate area of quantification. 3V, third ventricle (Bregma: −0.82). Scale bar: 100 *μ*m. Error bars reflect mean ± SEM. *P < 0.05 versus CL8.

Our laboratory has recently demonstrated that limiting nutrient availability in the first 3 weeks of life (by increasing the number of pups, in the crowded litter (CL) model) leads to extension of mean and maximal lifespan (Sun et al. 2009). By using mice from litters supplemented to 12 or 15 pups (CL12 and CL15) and comparing them to control litters limited to eight pups, we have also found that early life CL intervention has permanent effects on metabolic characteristics including reduced body weight, elevated insulin and leptin sensitivity, and changes in energy balance (Sadagurski et al. 2014). We considered it likely that restricting nutrients in the first 3 weeks of life might trigger persistent changes in the function of hypothalamic neurocircuits that regulate energy and glucose homeostasis (Bouret 2009). We therefore estimated mRNA expression of hypothalamic neuropeptides critically involved in energy and glucose balance. We found no significant differences between CL8 control and CL12 mice in the expression of mRNA for ARC neuropeptide orexigenic and anorexigenic genes *Agrp*, *NPY,* and *Pomc* at 4 or 24 weeks of age, in response to fasting. We did note, however, slight but significant reduction in *Agrp* expression in CL 15, as compared to control mice, tested at 24 weeks of age (Fig.1A).

Hypothalamic axonal projections change rapidly in the first 3 weeks of postnatal development in mice (Bouret 2009). We paid particular attention to the development of projections from the ARC to the paraventricular nucleus of the hypothalamus (PVH), because of its well-established importance in the neural control of energy balance (Elmquist et al. 2005). We analyzed the immunoreactivity of AgRP and *α*-MSH containing fibers in PVH (Fig.1B). Both parvo- and magnocellular regions of the PVH contained a high density of AgRP and *α*-MSH fibers. Quantification of the fiber density in the anterior PVH revealed significant increases in both AgRP and *α*-MSH fiber densities in CL12 and CL15 mice compared to controls at both 6 and 24 weeks of age (Fig.1B). AgRP and *α*-MSH fibers densities were similarly increased in the posterior part of the PVH in CL12 and CL15 mice at 24 weeks of age, (AgRP fiber density: 41.9 ± 1.0 and 44.9 ± 1.7 for CL12 and CL15 as compared to 33.0 ± 2.1 for CL8 (*P* < 0.01); *α*-MSH fiber density: 14.8 ± 1.2 and 15.2 ± 1.1 for CL12 and CL15 as compared to 10.38 ± 1.4 for CL8 (*P* < 0.05) and we detected no differences in the size of the ARC or PVH between groups (data not shown). Thus, early life nutrient restriction leads to a rapid and long-lasting increase in ARH fiber densities in neurons of the PVH that are critically involved in the regulation of energy balance and glucose homeostasis, with similar increases in both orexigenic and anorexigenic projections.

### No effect of caloric restriction after weaning on axonal projections of ARH neurons

**Figure 2:**
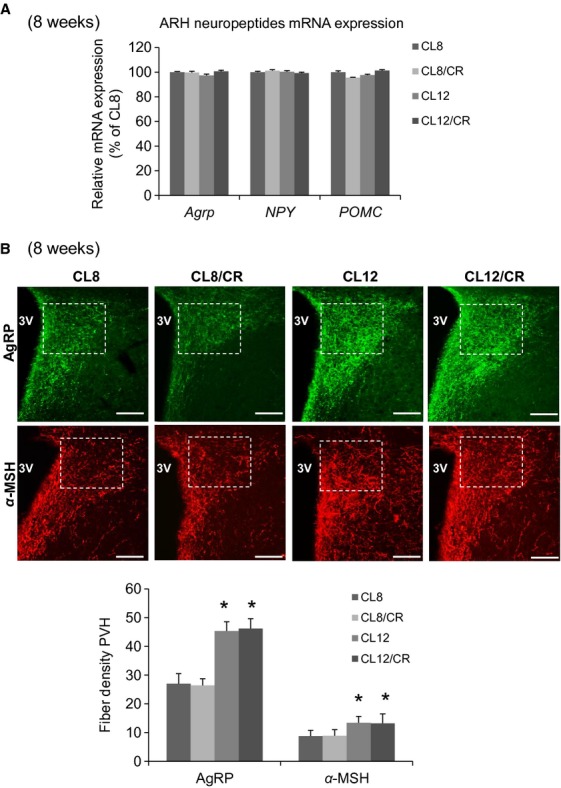
No effect of caloric restriction after weaning on axonal projections of ARH neurons. (A) Quantitative real-time PCR analysis of hypothalamic agouti-related peptide (*Agrp*), neuropeptide Y (*NPY*), and proopiomelanocortin (*POMC*) mRNA expression in 8-week-old CL8 (control) and CL12 female mice, and in CL8 and CL12 female mice subjected to an additional 3 weeks of caloric restriction (CL8/CR and CL12/CR) (*n* = 6/each group). (B) Images and quantification of agouti-related peptide (AgRP) and *α*-melanocyte-stimulating hormone (*α*-MSH) immunoreactive fibers innervating the paraventricular nucleus of the hypothalamus (PVH) at 8 weeks of age; (*n* = 6/each group). White boxes indicate area of quantification. 3V, third ventricle (Bregma: −0.82). Scale bar: 100 *μ*m. Error bars reflect mean ± SEM. *P < 0.05 versus CL8.

We also determined whether a further period of caloric restriction (CR), after weaning, would lead to additional changes in hypothalamic axonal projections in mice already exposed to the CL intervention. At weaning, subsets of CL12 and CL8 control mice were maintained for an additional 4 weeks on CR. mRNA expression for neuropeptides and densities of ARH neuronal fibers in PVH were then measured at 8 weeks of age. We saw no effects of additional postweaning weeks of CR on neuropeptide gene expression (Fig.2A) or on the fiber density of AgRP and *α*-MSH in the anterior PVH (Fig.2B). Similarly, fibers density of AgRP and *α*-MSH in the posterior part of the PVH were unchanged (data not shown). Interestingly, 4 weeks of postweaning CR did not affect the hypothalamic projections to the PVH of control mice that had not been subjected to CL prior to weaning, suggesting that the 3 weeks immediately after birth are particularly important for the development of hypothalamic projections in mice.

### Hypothalamic leptin signaling in adult CL mice

**Figure 3:**
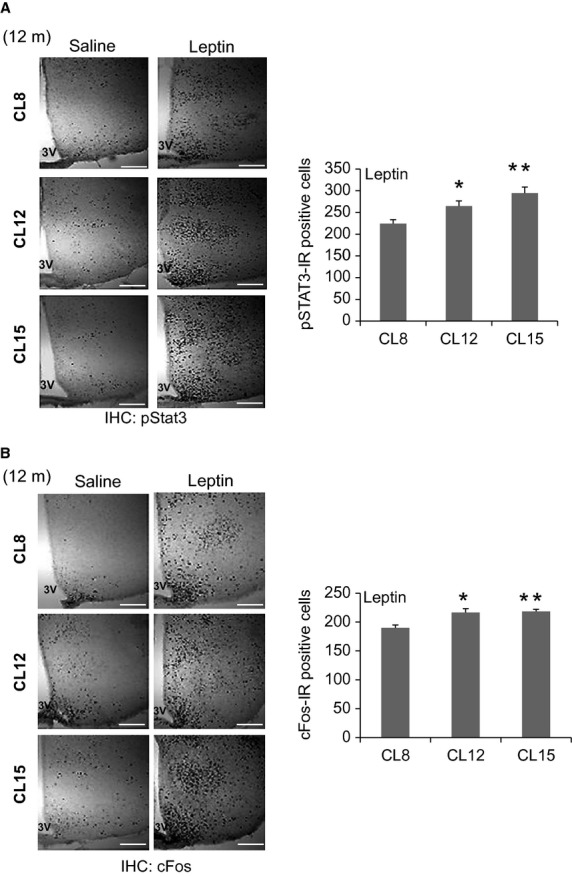
Hypothalamic leptin signaling in adult crowded litter (CL) female mice. Immunostaining for (A) pStat3 and quantification of pStat3 immunoreactivity and (B) cFos in 12-month-old female mice of the indicated groups 1 h after intraperitoneal injection of vehicle or leptin (5 mg/kg). 3V = third ventricle. Representative images from the hypothalamus are shown. Scale bar: 100 *μ*m. (*n* = 4/each group), error bars reflect mean ± SEM. *P < 0.05 versus CL8, **P < 0.01 versus CL8.

Our previous study demonstrated that CL mice are exceptionally leptin sensitive in adult life, as evaluated by changes in body weight and food intake after leptin injection at 6 months of age (Sadagurski et al. 2014). Leptin binding to LepRb activates an associated Jak2 tyrosine kinase, thereby promoting the phosphorylation of LepRb and the recruitment and tyrosine phosphorylation of Stat3 (signal transducer and activator of transcription-3) (Belgardt and Bruning 2010). To evaluate hypothalamic leptin responses, we measured leptin-stimulated accumulation of pStat3 in the brain, which reflects cell-autonomous LepRb signaling and is impaired in states associated with diminished leptin action (Munzberg et al. 2004). Acute leptin treatment led to an increase in levels of hypothalamic pStat3 in 12-month-old CL12 and CL15 female mice which was significantly greater (*P* < 0.05 and *P* < 0.01, respectively) than that seen in CL8 controls (Fig.3A). Similarly, leptin was more effective in CL12 and CL15 mice than in age-matched CL8 controls (*P* < 0.05 and *P* < 0.01, respectively) for stimulation of hypothalamic cFos-immunoreactivity, another measure of neuronal activation (Fig.3B). Thus transient, early food restriction in CL mice leads to long-lasting enhancement of hypothalamic LepRb signals, in addition to the effects on longevity.

### Hypothalamic insulin signaling in adult CL mice

**Figure 4:**
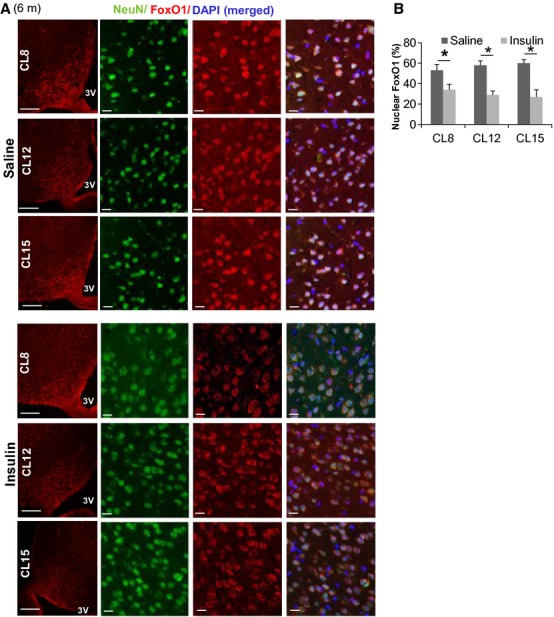
Hypothalamic insulin signaling in adult crowded litter (CL) female mice. (A) Immunofluorescence for FoxO1 (red) in 6-month-old CL8 (control), CL12, and CL15 female mice 1 h after intracerebroventricular injection with vehicle or insulin (300 mU). Representative images of the ARC from the hypothalamus of CL8, CL12, and CL15 mice are shown. Scale bars: 100 *μ*m (far left); 20 *μ*m (right side panels). The right side panels are magnified images representing area from the left panels, showing FoxO1 (red), NeuN (green), and merged with DAPI (blue) in 6-month-old mice of the indicated groups. (B) Quantification of neurons containing nuclear FoxO1 immunoreactivity (*n* = 4/each group). Error bars reflect mean ± SEM. *P < 0.05.

Insulin receptors (IRs) are highly expressed within the developing hypothalamus (Belgardt and Bruning 2010). Insulin or IGF-1 stimulation leads to the activation of the PI3K cascade, which inhibits FoxO1 action by phosphorylation-dependent export from the nucleus (Brunet et al. 2001). Our previous data demonstrated that CL mice have lower fasting insulin plasma levels compared to control mice (Sadagurski et al. 2014). To establish whether early life CL intervention affects hypothalamic insulin signaling, we investigated the distribution of FoxO1 between the nuclear and cytoplasmic compartments in ARC neurons of CL12, CL15, and CL8 control female mice at 6 months of age (Fig.4A). We identified neurons in these sections by immunostaining with NeuN and verified the location of the nucleus by DAPI staining (Fig.4A). Before insulin treatment, 50–60% of the ARC neurons contained nuclear FoxO1 in fasted control and CL mice. After insulin injection into the lateral ventricle, nuclear FoxO1 was detected in approximately 30% of the ARC neurons, but there were no significant differences between control and CL12 or CL15 mice (Fig.4B). These results suggest that hypothalamic insulin signaling is not disrupted by the CL intervention.

### Hypothalamic inflammation during aging in CL mice

**Figure 5:**
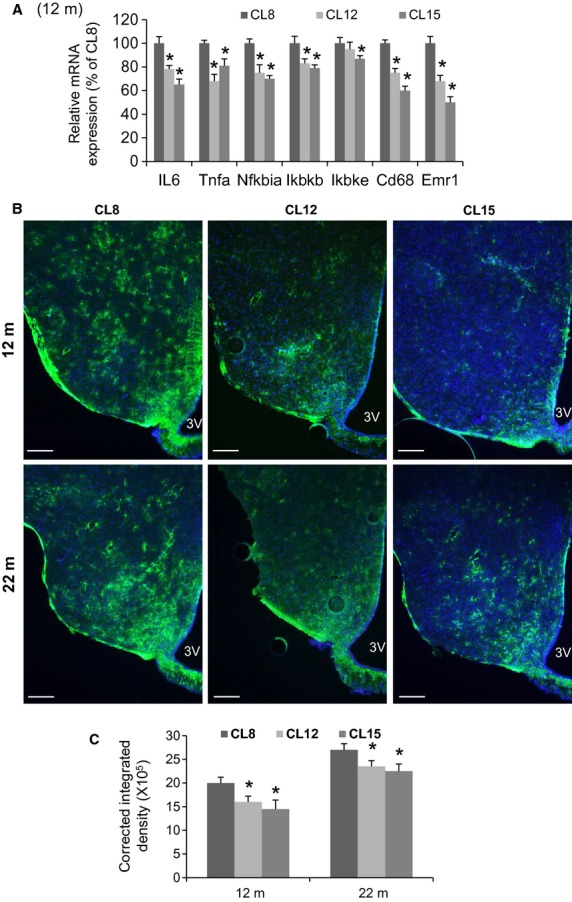
Hypothalamic inflammatory gene expression and astrogliosis during aging in crowded litter (CL) female mice. (A) Quantification of mRNA encoding proinflammatory cytokines (*Il6, Tnfa*), NF-*κ*B pathway genes (*Nfkbia, Ikbkb, Ikbke*), and microglia-specific (*Cd68* and *Emr1*) genes in the hypothalamus of 12-month-old CL8 (control), CL12, and CL15 female mice (*n* = 6/group), *P < 0.05 versus CL8. (B) Representative images of astrocytes identified by immunofluorescent detection of GFAP protein and DAPI (merged) in coronal sections of hypothalamus obtained from 6-week-, 12-month-, and 22-month-old female mice. Scale bar: 100 *μ*m. (C) Quantification of GFAP staining integrated density corrected for the background (error bars reflect mean ± SEM) in the region of the ARC from CL8, CL12, and CL15 female mice (*n* = 6/each group), *P < 0.05 versus CL8.

Inhibition of the hypothalamic inflammatory pathway slows down the aging process and increases mouse lifespan, suggesting that hypothalamic function may be of fundamental importance in the regulation of aging (Cai 2009). To assess the effect of age on hypothalamic inflammation in CL mice, we examined hypothalamic proinflammatory gene expression in middle-aged 12-month-old mice. Relative to CL8 control mice, gene expression analysis revealed reduced hypothalamic levels of *Il6*, *Tnfa*, *Nfkbia*, *Ikbkb*, and *Ikbke* mRNA in CL12 and CL15 female mice (Fig.5A). Furthermore, hypothalamic expression of mRNA encoding myeloid cell-specific markers *Cd68* and *Emr1* (which encodes F4/80) was also reduced compared to controls, suggesting an effect of CL on microglial accumulation in this brain area (Fig.5A). Astrogliosis with advancing age is correlated with increased expression of glial fibrillary acidic protein (GFAP) (Nichols et al. 1995). There is an age-related increase in levels of GFAP in 12- and 22-month-old controls and CL mice (compared to 6-week-old mice, data not shown), but the intensity of GFAP staining and the number of immunostained astrocytes in the ARC of CL mice is reduced by approximately 20–30% compared to CL8 control female mice at both 12 and 22 months of age (Fig.5B and C).

**Figure 6:**
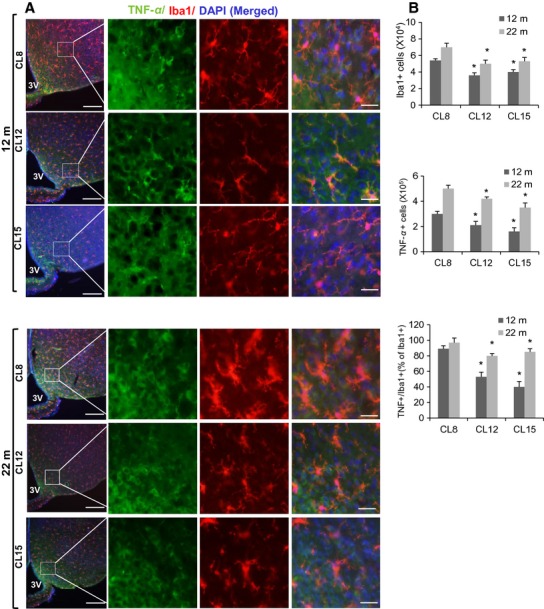
Hypothalamic inflammation during aging in crowded litter (CL) female mice. Brain sections of 12- and 22-month-old mice were analyzed for hypothalamic microglia and TNF-*α*. (A) Representative images immunostaining in MBH subregion of CL8, CL12, and CL15 mice are shown. Scale bars: 100 *μ*m (far left); 25 *μ*m (right side panels). (B) Numbers of cells immunoreactive for Iba-1, or TNF-*α* or the number cells positive for both TNF-*α* and Iba-1 as a percentage of Iba-1-positive cells in the hypothalamic mediobasal region (across the confocal microscopic field of serial sections) from CL8, CL12, and CL15 female mice (*n* = 6/each group), error bars reflect mean ± SEM *P < 0.01 versus CL8.

Next, we evaluated the numbers of microglia in the hypothalamus of aged CL mice. Using immunostaining for the microglia-specific Iba1 marker, we found that numbers of microglial cells in the mediobasal hypothalamus (MBH) increase in an age-dependent manner (Fig.6A). About 80–100% of these Iba1+ cells produced tumor necrosis factor-*α* (TNF-*α*) in control CL8 mice at 12 and 22 months of age, indicating that they are inflammatory (Fig.6A). In CL12 and CL15 mice, however, the proportion of Iba1 +  microglia that produce TNF-*α* is significantly reduced (*P* < 0.01) compared to age-matched CL8 controls at either age (Fig.6B). Hypothalamic *Tnfa* mRNA levels, evaluated at 12 months of age, are in good agreement with the TNF-*α* results (Figs.5A and 6B). Together, our data thus document long-term hypothalamic changes as a result of nutritional deprivation in the first few weeks of life, changes which may contribute to the longevity of CL mice.

## Discussion

Previous work has shown that mice subjected to litter crowding between birth and weaning are significantly longer lived than controls (Sun et al. 2009), have elevated expression of hepatic mRNA for enzymes involved in xenobiotic metabolism as late as 22 months of age (Steinbaugh et al. 2012), and differ from controls in obesity, glucose tolerance, and pancreatic beta cell mass (Sadagurski et al. 2014). The changes in responses to glucose, insulin, and leptin prompted us to evaluate the hypothalamic status of the CL mice, and here we have demonstrated changes in the organization of hypothalamic neural circuits controlling appetite, and enhanced hypothalamic leptin signaling, which persist many months into adult life. Our data are consistent with reports from other groups that have evaluated postnatal overnutrition or undernutrition through litter size adjustment and cross-fostering methods, which have shown that an altered milieu during the early postnatal period in rodents can override prenatal factors and genetic predisposition (Glavas et al. 2010). A report (Zhang et al. 2013) showing that alteration of hypothalamic inflammatory pathways can extend longevity in mice lends support to the idea that modulation of hypothalamic inflammatory response might contribute to the longevity of CL mice.

Arcuate nucleus of the hypothalamus neural circuits can be modulated by changes in insulin and leptin levels, which vary through juvenile and adult life (Bouret and Simerly 2006). For example, rearing rats in large litters enhances the development of the ARC-PVN pathway, with effects on leptin sensitivity (Patterson et al. 2010), consistent with our own CL mouse data. Plasma leptin levels are lower in CL mice from early adulthood (6 weeks of age) (Sadagurski et al. 2014), which may in turn contribute to the increased hypothalamic leptin sensitivity we report here. The effects of peripheral leptin levels during hypothalamic development have been well documented in earlier studies (Bouyer and Simerly 2013). We have shown that the CL intervention leads to equal enhancement of the opposing catabolic *α*-MSH and anabolic AgRP projections to the PVN. We hypothesize that this enhanced ARC-PVN pathway may reflect, or contribute to, the early increase in leptin sensitivity of the CL mice.

Adult caloric restriction (CR) results in reduced blood glucose levels and altered hormone levels, and these alterations are associated with longevity (Bartke et al. 2002). Reducing adipose stores is another way CR may act to alter circulating hormone levels (Ahima and Flier 2000). Among these, leptin and adiponectin are expressed by adipocytes differentially depending on adiposity and aging. Leptin is known to increase with adiposity (Frederich et al. 1995) and age (Ma et al. 2002). Maintenance of youthful adipokine levels by CR may prevent development of insulin resistance (Berg et al. 2001) and its consequences with aging. Because ARC neuropeptides are responsive to circulating insulin and leptin (Schwartz et al. 1992), ARC neuropeptides may be critical regulators of the effects of CR. In support of this idea, 6 weeks of adult CR leads to increase in AgRP and decreases in CART and POMC gene expression (Minor et al. 2009). In our CL mice, however, 4 weeks of continued CR after weaning had no further influence on expression of neuropeptide genes. In mice, projections of ARC axons involved in metabolic control develop after birth and remain both structurally and functionally immature until the second week of life (Bouret 2009). Similarly, maternal diet during lactation effects the establishment of ARH neuronal projections (Vogt et al. 2014). Our data also suggest that the first 3 weeks of life may be particularly important for development of hypothalamic circuitry, in that imposition of CR immediately after weaning had no significant effect on the hypothalamic projections or neuropeptide gene expression, either in CL mice or in control animals not previously subjected to litter crowding.

Postnatal insulin levels modify hypothalamic circuitry and development of ARC projections (Bouret 2009). Because CL mice are more glucose tolerant and insulin sensitive as they get older (Sadagurski et al. 2014), we hypothesized that enhanced hypothalamic insulin signaling might contribute to their phenotype, but we did not detect significant changes in hypothalamic insulin signaling in CL mice. Although insulin has been reported to have axonotrophic effects in vitro (Schechter et al. 1999), it has recently been suggested that insulin signaling is not essential for POMC axonal organization under normal developmental conditions (Vogt et al. 2014). Similarly, under obese conditions, activation of insulin signaling in the ventromedial nucleus of the hypothalamus contributes to the diet-induced inhibition of POMC neurons (Klockener et al. 2011). Development of hypothalamic neurocircuits may also be influenced by ghrelin, corticosterone, serotonin, or free fatty acids levels (Glavas et al. 2010; Bonnin and Levitt 2011; Sasaki et al. 2013), none of which have yet been evaluated in CL mice.

Activation, recruitment, and proliferation of microglia and astrocytes are hallmarks of the brain response to neuronal injury and could indicate the onset of physiological imbalances in the brain with age (Garcia-Caceres et al. 2013). The astrogliosis associated with advanced age is characterized by astrocyte hypertrophy and increased GFAP expression (a surrogate for astrocyte number). Increased numbers of GFAP-immunoreactive astrocytes were found in the hypothalamus of female C57BL/6J mice by 23 months (Kohama et al. 1995). mRNA for GFAP increased at later ages in brains of rodents and humans (Nichols et al. 1995). Chronic food restriction throughout adult life prevented an increase in hypothalamic GFAP mRNA levels by 24 months of age, but reached the same level as control rats by 33 months (Nichols et al. 1995). The relatively slow progression in the age-related increase in GFAP immunoreactivity in the hypothalamus of CL mice suggests that some age-related changes in astrocytes can be retarded by preweaning milk restriction.

The effect of inflammation on hypothalamic function is profound and includes alterations of homeostatic set points of multiple feedback loops (Cai and Liu 2011). We demonstrate that in long-lived CL mice, which are characterized by enhanced leptin sensitivity, hypothalamic inflammation is reduced throughout adult life, that is, at least between the ages of 12 and 22 months. Hypothalamic expression of TNF*α*, a proinflammatory cytokine, increases with age (Zhang et al. 2013), but is lower in CL12 and CL15 mice, compared to CL8 controls, at 12 or at 22 months of age. Similarly, hypothalamic levels of NF-*κ*B inflammatory signaling genes are reduced in CL12 and CL15 mice relative to CL8 control mice. Recent findings demonstrated that activity of NF-*κ*B increases in many regions of the brain in aging mice, but that this increase is greatest in the hypothalamus (Gabuzda and Yankner 2013). NF-*κ*B is an important regulator of gene transcription that mediates inflammatory responses, and has been implicated previously in the control of gene expression during aging (Cai 2009). Activation of NF-*κ*B in microglia stimulates secretion of the TNF-*α*, which, in turn, stimulates NF-*κ*B signaling in hypothalamic neurons (Cai 2009; Zhang et al. 2013). In CL12 and CL15 mice, microglia and TNF*α* activity are reduced compared to controls both at 12 and 22 months of age. Aging-related activation of NF-kB signaling in brain or hypothalamus can extend lifespan (Zhang et al. 2013) and our results support the idea that reduction in inflammatory pathways in the hypothalamus may contribute to the slow aging process of CL mice.

The conclusions of our study pertain to the properties of individual mice, and not to individual litters. There are some questions for which the litter would be the appropriate unit for analysis. Studies involving the influence of the nursing environment take the litter conditions into account, such as studies of the proportion of newborns surviving to weaning as a function of maternal genotype. In essentially all studies of mouse physiology, the set of mice used in the investigation include many animals which shared a mother, and these siblings are treated as individual units, Similarly, the conclusions of our study relate to properties of mice, not of litters, and we did not take the maternal influence into consideration. In summary, we report that the observed life extension in CL mice is associated with hypothalamic developmental changes and reduced hypothalamic inflammatory responses with age. Although the implications of our results to humans warrant caution, based on these developmental differences, our data indicate that aging might be postponed and lifespan extended by early life diet modifications. Collectively, this work identifies a strong link between early life caloric restriction and long-term changes in hypothalamic neurocircuitry and function in mice.
